# Influential nodes identification using network local structural properties

**DOI:** 10.1038/s41598-022-05564-6

**Published:** 2022-02-03

**Authors:** Bin Wang, Junkai Zhang, Jinying Dai, Jinfang Sheng

**Affiliations:** School of Computer Science and Engineering, Central South University, Changsha, 410083 China

**Keywords:** Diseases, Physics

## Abstract

With the rapid development of information technology, the scale of complex networks is increasing, which makes the spread of diseases and rumors harder to control. Identifying the influential nodes effectively and accurately is critical to predict and control the network system pertinently. Some existing influential nodes detection algorithms do not consider the impact of edges, resulting in the algorithm effect deviating from the expected. Some consider the global structure of the network, resulting in high computational complexity. To solve the above problems, based on the information entropy theory, we propose an influential nodes evaluation algorithm based on the entropy and the weight distribution of the edges connecting it to calculate the difference of edge weights and the influence of edge weights on neighbor nodes. We select eight real-world networks to verify the effectiveness and accuracy of the algorithm. We verify the infection size of each node and top-10 nodes according to the ranking results by the SIR model. Otherwise, the Kendall $\tau$ coefficient is used to examine the consistency of our algorithm with the SIR model. Based on the above experiments, the performance of the LENC algorithm is verified.

## Introduction

With the development of graph theory, the complex network has been applied in many fields^1–4^. Nodes and edges in different types of networks play diverse roles in network structure and function. These networks are heterogeneous at macro, mesoscale, and micro scales. At the macro level, we mainly focus on the statistical characteristics of networks, such as degree distribution, cluster distribution, and degree correlation. At the mesoscale level, the hierarchical and community structure of the network are the two most prominent characteristics. At the micro-level, the number of local neighbors nodes and the weight of edges is the focus of many scholars.

Traditional methods of identifying influential nodes mainly consider the degree of nodes, such as Hindex^5^, K-shell^6^, and Semi-local centrality^7^. In addition, the methods based on the influence of edges do not consider the different roles of edges in the process of information transmission, and the time complexity is high, such as Closeness centrality^8^ and Betweenness centrality^9^. Based on the mentioned above, many research works explore potential features in complex network from different perspective^10–13^. At present, many scholars have proposed methods to identify influential nodes^14–17^. For instance, to solve the limitation of the existing model in terms of research content is too single, Zhao et al.^18^ proposed a new method. It takes into account the influence of the node itself and its neighbor nodes. Also, a model to quantify the global influence of nodes are proposed, which makes the ranking more intuitive. Xu et al.^19^ designed two different influential nodes identification algorithms based on information entropy for four different types of networks. Consider the limited local information of the centrality method that may lead to incomplete identification of influential nodes, Maji et al.^20^ presented an improvement method that identifies the influential nodes even when the complete network structures are unavailable.

In recent years, many entropy-based centrality measures have been proposed. For instance, to design a more applicable centrality measure, Xu et al.^19^ proposed two influential nodes identification algorithms based on node adjacency information entropy (AIE). By calculating and comparing the adjacency information entropy of nodes, the importance of nodes is ranked. The larger the entropy value is, the more influential the nodes are. The algorithms highlight the different characteristics of the different types of networks (directed network and weighted network). To design a more robust and practical algorithm, Guo et al.^21^ proposed the EnRenew algorithm aimed to identify a set of influential nodes via information entropy (IE). Firstly, the information entropy of each node is calculated as the initial spreading ability. Then, select the node with the largest information entropy and renovate its *l*-length reachable nodes’ spreading ability by an attenuation factor, repeat this process until the specified number of influential nodes are selected. By taking the effect of the spreading rate on information entropy into account, Zhong et al.^22^ proposed an improved information entropy (IIE) method. The IIE method takes the spreading rate and the number of the target node’s neighbors into account. The above entropy-based algorithms for identifying influential nodes have all proved their accuracy through experiments. In the time complexity analysis section, we will compare the computational complexity of these algorithms with our proposed algorithm.

In this paper, considering the computational time of large-scale complex networks, we propose an algorithm that has low time complexity, can identify influential nodes with more accuracy. The algorithm can directly identify influential nodes without setting any parameters because some parameters are set to reasonable constants.

The rest of the paper is organized as follows. "Preliminaries" section gives a brief introduction to the preliminaries. "Methods" section presents the LENC ranking algorithm we proposed, including the main idea, and the calculation process of our algorithm. "Experiments" section will point out the experimental verification. "Discussion" section is the conclusion.

## Preliminaries

A network can be denoted by *G*, equated as $G = (V, E)$, where *V* and *E* represent the set of nodes and edges, respectively.

### Equilateral triangle

The edge between node $V_{m}$ and $V_{n}$ is expressed by $E_{mn}$. Assuming that the neighbor node sets of $V_{m}$ and $V_{n}$ are $\Gamma (V_{m})$ and $\Gamma (V_{n})$, respectively, then the number of triangles that can form between the two nodes is the number of their common neighbors. It can be defined as

1 $$\begin{aligned} Triangle(E_{mn}) = \parallel {\Gamma (V_{m})}\cap {\Gamma (V_{n})}\parallel . \end{aligned}$$

### Edge weight

On the one hand, the more paths connected by the node, the greater the information load, and the greater the influence of corresponding edges. On the other hand, the more alternative paths are available, the influence of the edge will be reduced correspondingly^23^. Besides, the contribution of the edge to the information transmission is proportional to the information load of the node. Based on the above considerations, the influence of an edge depends on the information-carrying capacity of the connected nodes and the possibility of the edge is being replaced by other paths. The weight of edge $E_{mn}$ between node $V_{m}$ and $V_{n}$ can be expressed as

2 $$\begin{aligned} Weight(E_{mn}) = \frac{(k(v_{m})-T_{mn})(k(v_{n})-T_{mn})R_{mn}w_{mn}}{(T_{mn}/2)+1}, \end{aligned}$$

3 $$\begin{aligned} R_{mn} = \frac{k(v_{m})}{k(v_{m})+k(v_{n})}, \end{aligned}$$

where $k(v_{m})$ represents the degree of node $v_{m}$, $T_{mn}$ represents the number of triangles formed by the edge $E_{mn}$, $w_{mn}$ represents the weight of the edge between node *m* and *n*, and $R_{mn}$ represents the contribution coefficient of the edge. For simplicity, we set $w_{mn}=1$. $Weight(E_{mn})$ is abbreviated as $W_{mn}$ in this paper. Since the contribution of edge to the influence of the two nodes it connects is different, the same edge reflects different influences to the two nodes, expressed as $W_{mn}\ne W_{nm}$.

The virtual node $V'$ and other nodes in the network have a virtual edge $E_{mv'}$, and the number of triangles constituted by the virtual edge is assumed to be 0 in the algorithm. The weight calculation method of the virtual edge is the same as that of other edges, and the weight of edge $E_{mv'}$ is expressed as

4 $$\begin{aligned} W_{mv'} = k(v_{m})k(v')\frac{k(v_{m})}{k(v_{m})+k(v')}=\frac{k(v_{m})^2 k(v')}{k(v_{m})+k(v')}, \end{aligned}$$

where $k(v')$ represents the information load (degree value) of the virtual node. Since the virtual node connected to all nodes in the network, here $k(v') =N$, and *N* is the number of nodes in the network.

The influence of all edges around the node is added, the sum of the weights of the first order edges of the nodes can be expressed as

5 $$\begin{aligned} W_{m} = W_{mv'} + \sum _{V_{n}\in \Gamma (V_{m})} W_{mn} , \end{aligned}$$

where $W_{m}$ represents the sum of the weights of all edges connected to the node $V_{m}$.

### Information entropy

Claude Shannon^24^ pointed out that information entropy is monotone, non-negative and additive. The only form of the uncertainty measurement function of a random variable that has proved to satisfy the three conditions is $H(X) = - C \sum P(X)\log _{2}{P(X)}$, which is suitable for constant *C*, in this case, we set $C=1$. The entropy of the node based on the weight distribution of the edges connected to it of virtual node and neighbor node defined as

6 $$\begin{aligned} Entropy(E_{mn})= & {} -\frac{W_{mn}}{W_{m}}\log _{2}\frac{W_{mn}}{W_{m}}, \end{aligned}$$

7 $$\begin{aligned} Entropy(E_{mv'})= & {} -\frac{W_{mv'}}{W_{m}}\log _{2}\frac{W_{mv'}}{W_{m}}. \end{aligned}$$

The sum of the information entropy of all first-order edges of a node is obtained by summing up the information entropy of all first-order edges of a node, which is similar to the method of calculating and evaluating the influence of nodes based on degree entropy^25^. In this algorithm, the entropy of the node based on the weight distribution of the edges connected to it is used to evaluate the influence of nodes, and the edge entropy weight of nodes is expressed as

8 $$\begin{aligned} Entropy(V_{m}) = Entropy(E_{mv'})+\sum _{v_{n}\in \Gamma (v_{m})} Entropy(E_{mn}). \end{aligned}$$

### Node influence

According to the topological structure of the network, nodes at the center of the network have a higher influence than nodes at the edge of the network. In the case of two nodes with the same entropy value, the node at the center is more important than the node at the edge. When calculating the local influence of nodes, the position influence coefficient k-core is introduced. The first-order entropy of the node based on the weight distribution of the edges connected to the node is the contribution of the first-order edge to the node influence, which is expressed as

9 $$\begin{aligned} influence(v_{m}) = Entropy(v_{m}) k-core(v_{m}). \end{aligned}$$

To ensure the accuracy of the algorithm, the second-order edge entropy should be considered. The first-order edge is the entropy of the node based on the weight distribution of the edges connected to the node itself, and the second-order edge is the entropy of the node based on the weight distribution of the edges connected to the neighbor nodes, which is the contribution of the neighbor to the influence of the node^26^. The total influence of nodes in the network can be expressed as

10 $$\begin{aligned} Influence(v_{m}) = influence(v_{m}) + \sum _{v_{n} \in \Gamma (v_{m})} influence(v_{n}), \end{aligned}$$

where, $v_{n}$ are the neighbor nodes of node $v_{m}$.

## Methods

### Main idea

According to the properties of information entropy, information entropy can measure the uncertainty of the system. The more stable the system is, the higher the information entropy is. Otherwise, the information entropy is lower. Therefore, the entropy of the node based on the weight distribution of the edges connected to it can be used as an indicator to evaluate the local influence of nodes. The higher the entropy is, the higher the complexity of nodes is, and the more influential it is in the network. There are two extreme cases of ranking the influence of nodes by using information entropy: the entropy value of nodes with one edge is 0, and the entropy value of nodes with a similar structure is equal.

**Figure 1:**
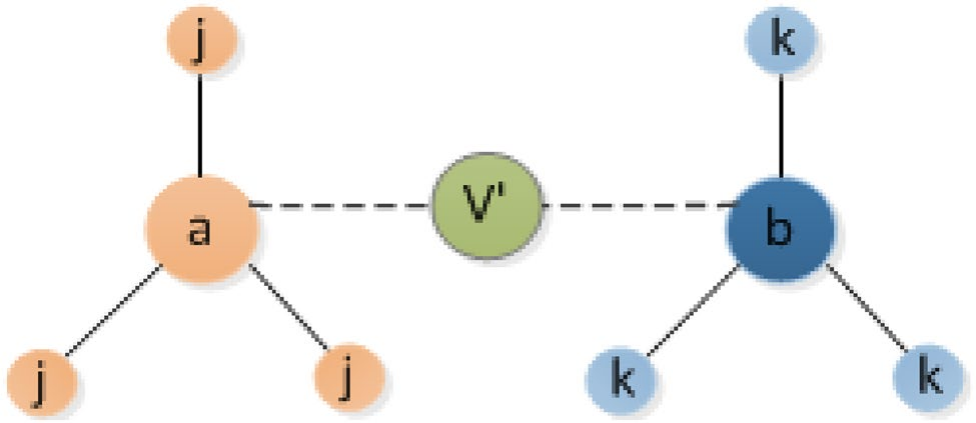
Virtual node action display diagram.

As shown in Fig. 1, node *a* and node *b* in the network have the same number of neighbor nodes. It is assumed that the degree of the node *j* and *k* are not equal, the weights are different but the distribution is the same. If we calculate the information entropy by the difference distribution, the weighted entropy of both nodes are the same, and the value is $E=-\log _{2}{(1/3)}$. Hence the influence is indistinguishable. However, with the introduction of the virtual node $V'$, the weight and the connecting edge of the virtual node to the two nodes are the same, without changing the network attribute. The weight value of the virtual edge and real edge is different due to the difference of nodes, which breaks the original balanced distribution so that the influence of the two nodes can be well distinguished. The influence of nodes can be obtained by the entropy weight of multi-order edge information.

### Information entropy model

Introducing virtual nodes to reconstruct the network, assigning edge weights to nodes, and calculating the entropy of the node based on the weight distribution of the edges connected to it, the contribution of edges to the influence of nodes is determined. On this basis, introduce the location of the nodes in the network parameters, ensure the rationality of the proposed algorithm. The calculation considers the two-layer edge of the node to ensure the accuracy and efficiency of the algorithm. We mainly introduce the construction process of the LENC algorithm model from three aspects: algorithm definition, algorithm flow, and time complexity analysis.

### The algorithm flowchart

**Figure 2:**
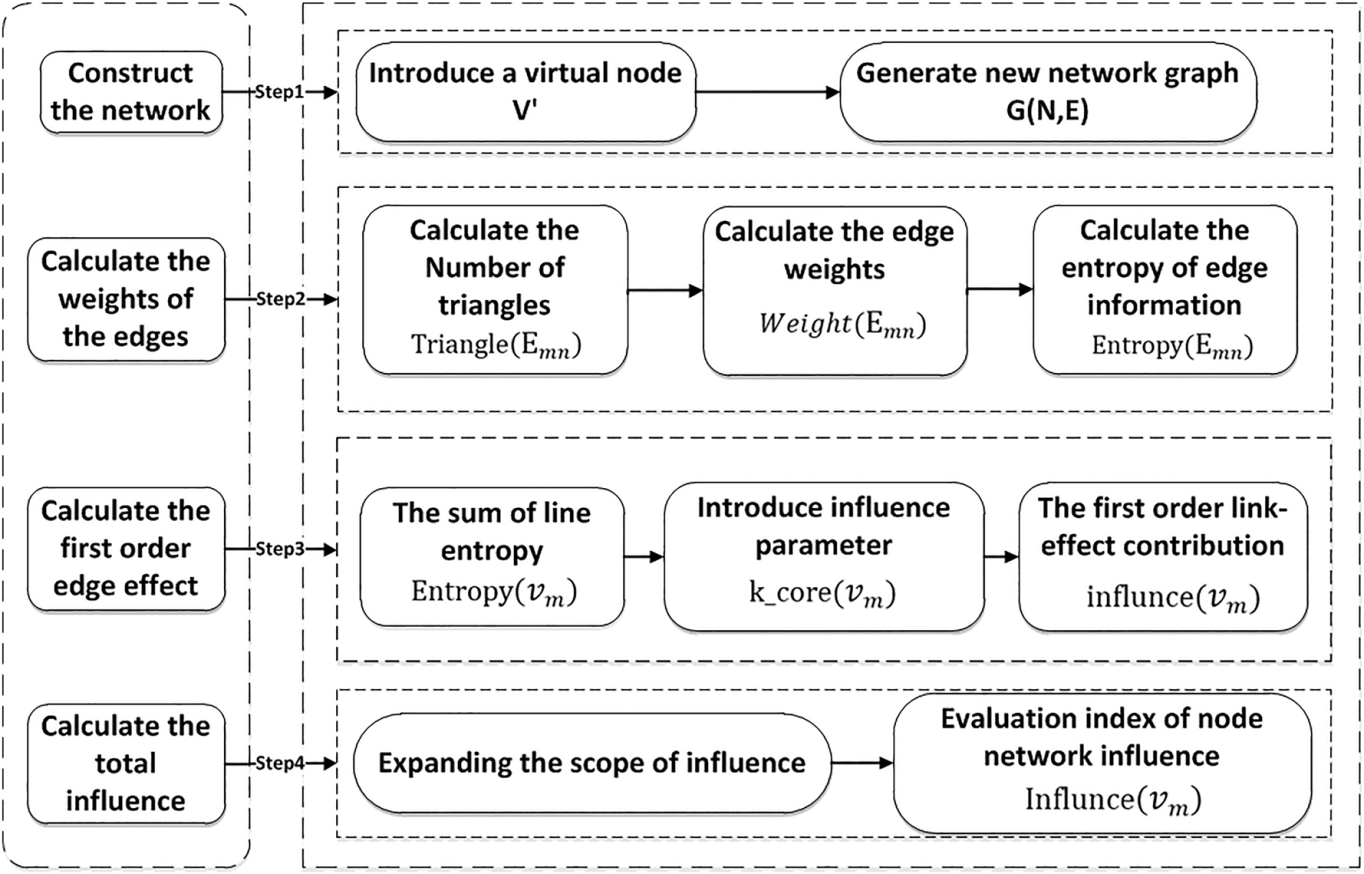
The flow chart of the LENC algorithm.

The idea of the LENC algorithm is as follows. Firstly, a virtual node $V'$ is introduced to reconstruct the network, forming a new network $G=(V, E)$. Node $V'$ has a virtual edge with all nodes in the network, and the degree of node $V'$ is the total number of nodes in the network. Secondly, according to the number of adjacent triangles and the effect of the nodes on the influence of adjacent triangles, the weights of adjacent nodes and neighbor nodes, and virtual nodes are calculated. Then, the entropy value of each edge is obtained according to the information entropy formula, and the entropy value of the first-order edge of the node to obtain the local influence of the node. Finally, the local influence attributes of the neighbor nodes are added to obtain the entropy weight of the first and second-order edges of the nodes, which can be an indicator of the influence ability of the nodes in the network. Figure 2 shows the calculation process of the model.

### Time complexity

The time complexity of the LENC algorithm has three main components. In the first part, to calculate the weight of the edge, we need to consider the number of common nodes among the nodes and their neighbors. First, calculate the number of the triangle of the edge, then calculate the weight of the edge. The time complexity is *O*(*N*), where is the average degree of the network, and N is the number of nodes; The second part is to calculate the local influence of nodes, which requires the introduction of the location attribute k-core of nodes. According to the K-shell algorithm, this step requires the traversal of all edges in the network. The time complexity is *O*(|*E*|), where E is the number of edges. In the third part, to calculate the total influence of the node, it is necessary to accumulate the weighted entropy of the first and second-order edges of the node. To calculate the weighted entropy of the neighbor edges, it is necessary to continue to traverse two-layer neighbor nodes, with the time complexity of $O(N<k>^2)$. Therefore, the time complexity of the LENC algorithm is $O(N<k>^2+|E|)$. Table 1 lists the time complexity of several state-of-the-art algorithms and some popular entropy-based centrality measures. We can see the time complexity of LENC is low.

**Table 1:** Time complexity of different algorithms.

Algorithm	Complexity
CC	$O(n^2logn+nm)$
EC	$O(n^2)$
HITS	*O*(*n*)
Hindex	*O*(*nlogn*)
DIL	$O(n<k>^2)$
LENC	$O(n<k>^2)$
IIE	*O*(*n*)
AIE	$O(n^2)$
IE	$O(m+n+rlogn+\frac{rm^2}{n^2})$

(*n*, *m* and *r* represent the number of nodes, edges and initial infected nodes, respectively.)

### Computation process

**Figure 3:**
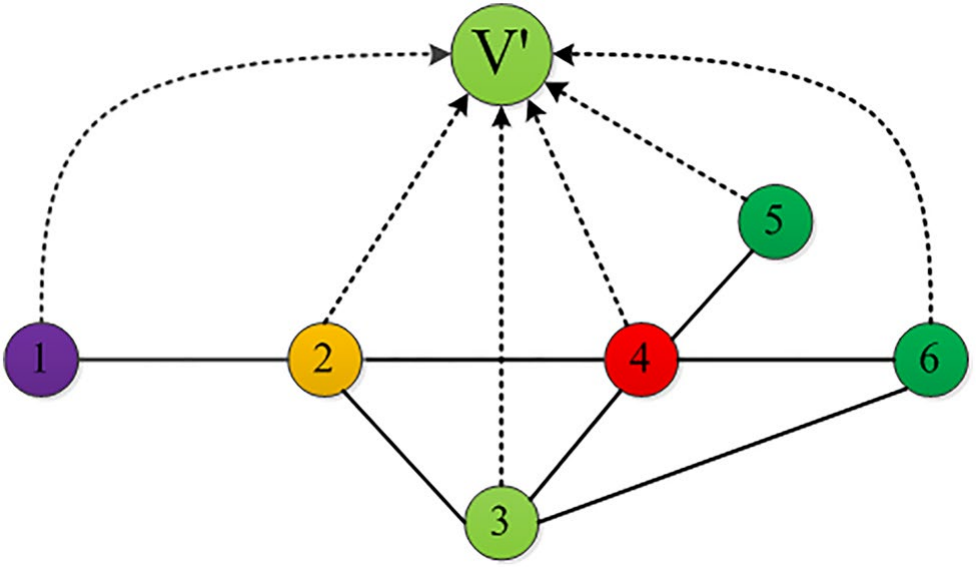
A toy network.

To further explain the specific calculation process of the LENC algorithm, a simple network that contains 6 nodes and 7 edges is an example. Node $V'$ is a virtual node introduced in the network. As shown in Fig. 3, take the influence of node $v_{4}$ in the network as an example. By calculating the entropy weight contribution of the first-order and second-order edges, the influence of the nodes was obtained. The specific calculation steps are as follows.

**Step 1: Calculate edge weight**. Calculate the weight of the edge between node $v_{4}$ and virtual node $V'$,

$$\begin{aligned} W_{4v^{'}}= \frac{k(v_{4 })^2\times k(v^{'})}{k(v_{4})+k(v^{'})}= (\frac{4^2\times 6}{4+6})= 9.6. \end{aligned}$$

Calculate the weight of the edge between node $v_{4}$ and neighbor node $v_{2}$,

$$\begin{aligned} Weight(E_{42})= \frac{(k(v_{4})-T_{42})\times (k(v_{2})-T_{42})}{(T_{42}/2)+1}\times R_{42}= \frac{(4-1)\times (3-1)}{(1/2)+1}\times (\frac{4}{4+3}) = 2.2857. \end{aligned}$$

In the same way, the weights of the edge of the neighbor node $v_{3}$, node $v_{5}$, and node $v_{6}$ can be calculated as follows: 0.5714, 3.2, 1.3333.

**Step 2: Calculate total weight**. Add the edge weights of node $v_{4}$ and all neighbors to obtain the first-order edge weights of node $v_{4}$,

$$\begin{aligned} Weight_{4}= W_{4v^{'}} + \sum _{V_{n} \in \Gamma (V_{4})} W_{4n} = 16.9904. \end{aligned}$$

**Step 3: Calculate the entropy value**. The entropy weights of the edges of node $v_{4}$ and its neighbors and virtual nodes are calculated respectively. Take the information entropy of the edges of node $v_{2}$ and its neighbors as an example,

$$\begin{aligned} Entropy(E_{42})= -(\frac{W_{42}}{W_{4}} )\log _2(\frac{W_{42}}{W_{4}})= -(\frac{2.2857}{16.9904}) \log _2(\frac{2.2857}{16.9904})=0.3893. \\ Entropy(E_{4v^{'}})= -(\frac{W_{4v^{'}}}{W_{4}}) \log _2(\frac{W_{4v^{'}}}{W_{4}})= -(\frac{9.6}{16.9904}) \log _2(\frac{9.6}{16.9904})=0.4654. \end{aligned}$$

Following this method, the entropy weights of the edges of node $v_{4}$ and all neighbor nodes are added (including the entropy values of the virtual node $V'$), so that the sum of the entropy values of all first-order edges of the node is

$$\begin{aligned} Entropy(v_{4})= Entropy(E_{(4v^{'})} )+ \sum _{v_{n}\in \Gamma (v_{4})}{Entropy(E_{4n})}= 1.76105. \end{aligned}$$

**Step 4: Calculate the total influence**. Combined with the location parameters of node, the local influence of nodes in the network is calculated, as shown in the following formula,

$$\begin{aligned} influence(v_{4})= Entropy(v_{4} )\times k-core(v_{4}) =1.76105\times 2=3.5221. \end{aligned}$$

According to the “three-degree separation” theory^27^, edges outside the third-order have less impact on the influence of nodes and even have a negative effect. To ensure accuracy, the second-order edges were considered. In addition to its local influence, the ultimate influence of nodes in the network should be extended to other neighbor nodes. The total influence of node $v_{4}$ in the network is calculated as follows.

$$\begin{aligned} Influence(4_{m})=influence(v_{4})+ \sum _{v_{n}\in \Gamma (v_{4})}{influence(v_{n})}= 12.8902. \end{aligned}$$

In the same way, we can calculate the influence of all nodes in the network. The results are shown in Table 2.

**Table 2:** Comparison result of simple network node influence evaluation indexes.

Node	4	3	2	6	5	1
k-core	2	2	2	2	1	1
Result	12.8902	11.8911	10.5953	8.4408	4.5212	4.4470

Next, we analyze the influence of nodes in the network according to the node deletion method. Firstly, nodes 3 and 4 are at the center of the network. The degree of node 4 is greater than that of node 3, and removing node 4 has a greater impact on the network structure. Therefore, the influence of node 4 is greater than that of node 3. Secondly, for node 2 and node 6, moving node 2 will cause the entire network to be disconnected, which has a greater impact on the network structure, so node 2 is more important than node 6. Finally, the influence of node 1 and node 5 at the edge of the network is similar. Therefore, according to the node deletion method, the ranking results of the influence of nodes are 4, 3, 2, 6, 5, 1. As shown in Table 2, the ranking results of our proposed algorithm are consistent with the analysis result. Therefore, the accuracy of LENC has been proved initially.

## Experiments

### Data sets

In this experiment, eight real-world networks with different properties are selected, the statistics of these networks are summarized as follows. The basic statistics are shown in Table 3, and these networks can be downloaded from KONECT (http://konect.uni-koblenz.de/networks/) and NETWORK (http://networkrepository.com/).

**Table 3:** The statistics of eight real-world complex networks: Node number |*V*|, edge number |*E*|, average degree $<K>$, maximum degree $K_{max}$, and clustering coefficient $<CC>$.

Data Sets	|*V*|	|*E*|	$<K>$	$K_{max}$	$<CC>$
Zachary	34	78	4.5882	17	0.5706
Arenas-email	1133	5451	9.62	71	0.2202
Moreno-blogs	1224	16715	27.312	351	0.3197
Web-spam	4767	37375	15.681	477	0.2859
Bio-dmela	7393	25569	6.916	17	0.5706
Ca-astroph	18771	198050	21.34	236	0.677
Email-EU	32430	54397	3.3547	623	0.1127
Opsahl-powergrid	4941	6594	2.669	19	0.0801

#### Zachary

The Karate club network, a total of 34 nodes and 78 edges. The nodes represent the club members, and the edges represent the bond between two club members.

#### Arenas-email

This is the E-mail network of Rovira I Virgili University in Tarragona, southern Catalonia, Spain. It consists of 1133 nodes and 5451 edges. In the network, the nodes represent e-mail users, and the edges represent at least one e-mail message that has been sent between two users.

#### Moreno-blogs

The Blog network contains hyperlinks on the front pages of blogs in the context of the 2004 USA election. A node represents a blog, and an edge represents a reference relationship between two blogs. There are 1224 nodes and 16715 edges in the network.

#### Web-spam

The network is provided by the Purdue university network repository and contains 4767 nodes and 37375 edges.

#### Bio-dmela

In biological networks, the nodes are proteins, and the edges are interactions between proteins. The nodes are individual proteins with a total of 7393 nodes. The edges represent the interactions between proteins with a total of 25569 edges.

#### Ca-AstroPh

The network is a collaboration diagram of authors of selected scientific papers in astrophysics (Astro-ph). Nodes represent each paper author, and edges indicate that the authors quote each other or have a cooperative relationship with each other. It contains 18771 nodes and 198050 edges.

#### Email-EU

The Email-EU network, containing 32000 nodes and 54000 edges, nodes represent all kinds of e-mail, and edges represent the interconnections among e-mail.

#### Opsahl-powergrid

This undirected network contains information about the power grid of the Western States of the United States of America. An edge represents a power supply line. A node is either a generator, a transformer, or a substation. It consists of 4941 nodes and 6594 edges.

### Comparison algorithm

Five comparison algorithms are selected in our experiment. They are described as follows.

#### CC (Closeness Centrality)

Closeness centrality is based on the global information of the network to determine the network influence of nodes. The smaller the relative distance between all the node pairs, the stronger the accessibility of node information, and the more important are the nodes. It has been widely used in research but the time complexity is high.

#### EC (Eigenvector Centrality)^28^

This method considers that the influence of nodes in the network depends on both the number of neighbor nodes and the influence of neighbor nodes themselves. Its essence is to increase the influence of the node itself by connecting other nodes of relative influence. However, when there are many nodes with a large degree in the network, the phenomenon of fractional convergence will occur^29^.

#### HITS

HITS algorithm using different metrics to assess the influence of the nodes in the network. Give each node a hub value and an authority value to evaluate the influence of the node. Authority value measures the original creativity of nodes to information, and hub values reflect the role of nodes in information transmission. They interact and converge iteratively.

#### Hindex

This algorithm is mainly used to evaluate a scholar’s academic achievements. The higher Hindex value indicates the greater influence of the node.

#### DIL

DIL is a new algorithm^23^. The method considers the degree attribute of the node but also the edge attribute of the node.

### Evaluation indicators

#### SIR model

Kermack and McKendrick proposed the SIR model in 1927^30^. The model includes S, I, and R states. S indicates susceptible, I state indicates infected, and it can infect other healthy nodes with a certain probability. The R indicates recovered and has immunity. The SIR model is defined as follows.

11 $$\begin{aligned} \left\{ \begin{array}{lr} \frac{ds(t)}{d(t)} = -\beta s(t)i(t) &{} \\ \frac{di(t)}{d(t)} = \beta s(t)i(t) - \gamma i(t) \qquad , &{} \\ \frac{dr(t)}{d(t)} = \gamma i(t) &{} \end{array} \right. \end{aligned}$$

where *S*(*t*), *I*(*t*) and *R*(*t*) represent the number of susceptible nodes, infected nodes, and recovered nodes at time *t* respectively. $\beta$ represents the probability of infection and $\gamma$ represents the probability of recovery.

### Kendall coefficient

Kendall $\tau$ coefficient^31^ is used to explain the correlation of two sequences, the correlation coefficient can reflect the proximity of two sequences. Suppose two sequences are related and have the same number of elements, expressed as $X =(x_{1}, x_{2}..., x_{n})$, $Y =(y_{1}, y_{2}..., y_{n})$. For the elements in both sequences, if $x_{i} > x_{j}$, $y_{i} > y_{j}$ or $x_{i} < x_{j}$, $y_{i} < y_{j}$, then any pair of sequence tuples $(x_{i}, y_{i})$ and $(x_{j}, y_{j})$, $(i\ne j)$ are considered to be concordant; If $x_{i} < x_{j}$, $y_{i} > y_{j}$ or $x_{i} > x_{j}$, $y_{i} < y_{j}$, they are considered discordant; If $x_{i}= x_{j}$ or $y_{i}= y_{j}$, they are considered neither consistent nor inconsistent. Kendall $\tau$ coefficient is defined as

12 $$\begin{aligned} \tau (X,Y) =\frac{n_{c} - n_{d}}{0.5n(n - 1)}, \end{aligned}$$

where *n* is total combinations in these sequences, ${n_{c}}$ and $n_{d}$ indicate the number of concordant and discordant pairs, respectively. It reflects the correlation and matching between two sequences. In general, $\tau \in [-1,1]$, where $\tau >0$ indicates a positive correlation and $\tau <0$ illustrates negative correlation. That is, the higher the $\tau$ value is, the more accurate the ranking.

### Experimental analysis

To verify the ability of the LENC algorithm to identify influential nodes, the SIR model and Kendall correlation coefficient are used as evaluation indicators and compare the accuracy and effectiveness of different algorithms.

### Case test

**Figure 4:**
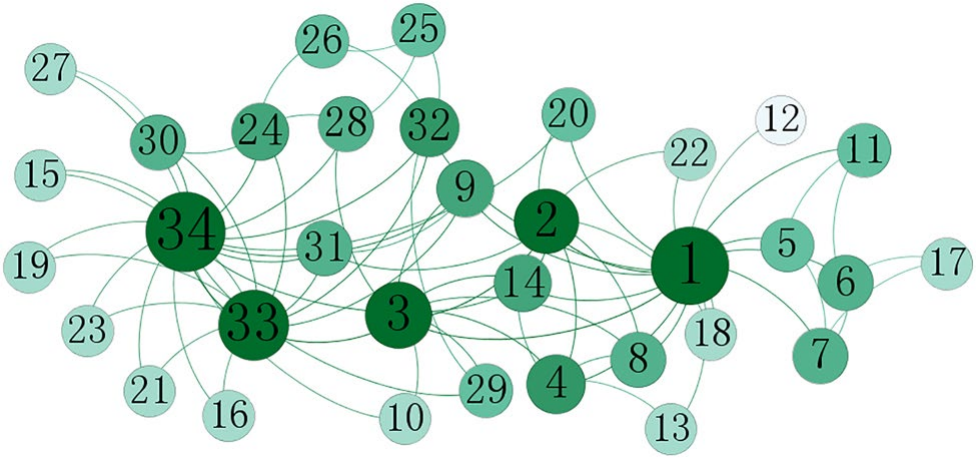
The karate network.

First, take the karate network as an example. The topology of the karate network is shown in Fig. 4. Table 4 shows the top-10 nodes ranking results of different algorithms and the SIR model. It can be seen from Table 4 that the ranking results of CC, Hindex, and DIL algorithms are different from the SIR model, which indicates that the ranking results of these three algorithms in the Zachary network are not accurate enough. The ranking results of the LENC, EC, and HITS algorithms are consistent with the SIR model, which can identify the influential nodes in the network accurately. Therefore, the accuracy of the LENC algorithm is proved preliminarily.

**Table 4:** Comparison of ranking results of top-10 nodes in karate network.

Rank	CC	EC	HITS	Hindex	DIL	LENC	SIR	SIR Value
1	1	34	34	1	34	34	34	3.58
2	3	1	1	3	1	1	1	3.31
3	34	3	3	14	3	3	33	3.00
4	32	33	33	33	33	33	3	2.94
5	9	2	2	34	32	2	2	2.65
6	14	9	9	9	2	9	9	2.34
7	33	14	14	2	14	32	4	2.31
8	20	4	4	24	28	14	14	2.30
9	2	32	32	31	9	4	32	2.24
10	4	31	31	4	24	31	31	2.05

### Correlation analysis

**Figure 5:**
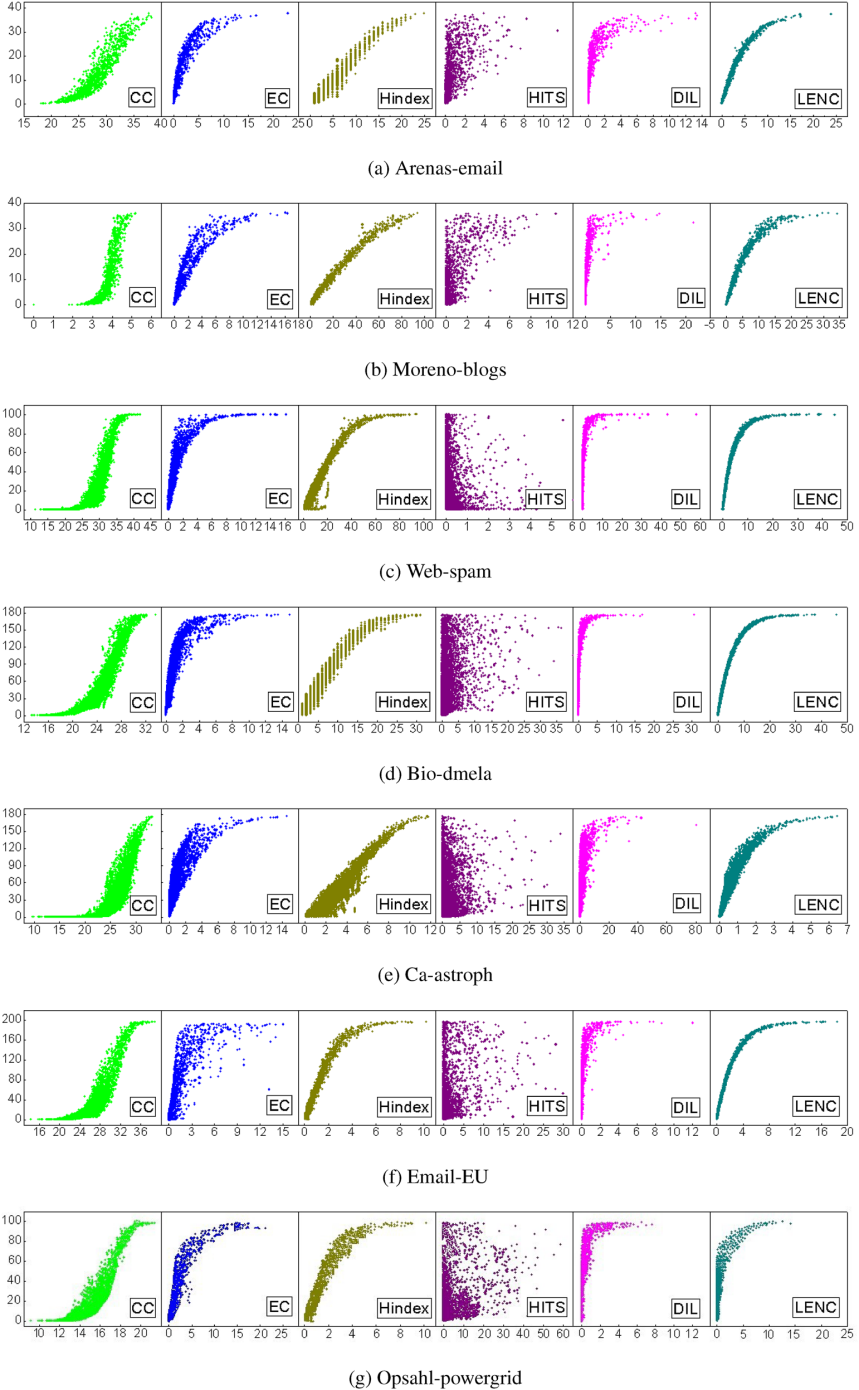
Correlation between significance evaluation indicator of different algorithms and SIR model infection.

In this experiment, the SIR model was used to evaluate the rationality and correctness of different algorithms. The infection probability^32^ is set as $\beta = 2<k>/<k^2>$, *k* represents the average degree of nodes in the network, and $<k^2>$ represents the second-order neighbor degree with recovery probability $\gamma =1$, running independently for 1000 times. Figure 5 shows how the number of infected nodes varies with the influence of nodes. The x-axis represents the influence of nodes in different algorithms, and the y-axis represents the average number of infected nodes corresponding to different infection probabilities. The more linear the curve is, the more accurate the ranking result is. To observation, the axis is scaled, as shown in Fig. 5. In the Arenas-email network, the linear growth trend of the LENC algorithm is obvious, which indicates that there is a positive correlation between the node influence and the SIR model. CC, EC, and Hindex algorithms perform well, but they can not accurately distinguish nodes with the same influence. In the HITS algorithm, the distribution of nodes is loose, and the influence of nodes in the same location is significantly different, which indicates that the algorithm is coarse-grained. In the Moreno-blogs network, the HITS algorithm performs worst. In Web-spam network, Bio-dmela, Opsahl-powergrid, and Email-EU network, the LENC algorithm is better than other algorithms because it has a significant positive correlation with the SIR model. Therefore, the LENC algorithm is suitable for different networks, and the ranking result of node influence is more accurate and reasonable. As the above result, the LENC algorithm has the best positive correlation with the SIR model. As the influence evaluation index increases gradually, the number of infected nodes in the SIR model increases steadily. Moreover, the number of nodes with the same influence is relatively concentrated, which indicates that this method can rank the influence of nodes more precisely.

### Transmission capacity

**Figure 6:**
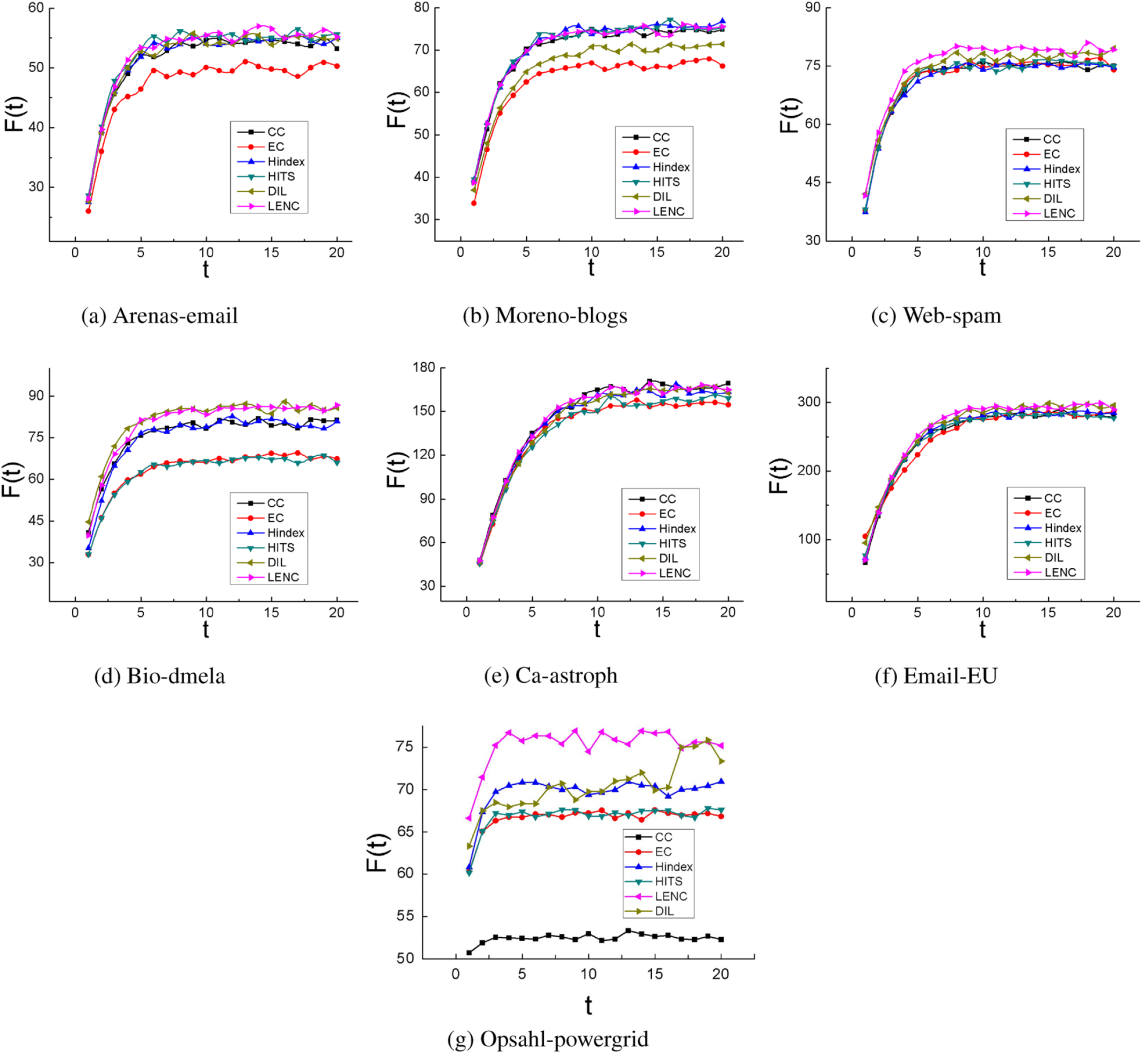
Transmission initial infection capacity of the top-10 nodes.

In this experiment, the top-10 nodes detected by different algorithms are used as infected nodes, and the number of infected nodes in each time step is used to distinguish the influence of nodes. To verify the initial infection ability of each algorithm, we set the infection probability $\beta = 0.01$, and the recovery probability $\gamma =1$. T is the time step, and *F*(*t*) represents the number of infected nodes in the network in time step t, as shown in Fig. 6. In the Web-spam network, the infected nodes of the top-10 nodes of the LENC algorithm are greater than other algorithms, indicating that the LENC algorithm can more identify the influential nodes in the network accurately. In the Bio-dmela and Email-EU network, the infection effect of the LENC and DIL algorithms is the best. In Arenas-email, Moreno-blogs and Ca-astroph networks, the ranking result of CC, Hindex, HITS, and LENC algorithms are similar. It is worth noting that the infection curve of the LENC algorithm has better infection performance. Besides, the ranking results of influential nodes identified by the EC algorithm do not meet the expected results. In summary, the positive correlation between the number of nodes infected by the LENC algorithm and the SIR model is the most obvious and verified the accuracy of this method. In the Opsahl-powergrid network, it can be seen from Fig. 6 that the infected performance of the LENC algorithm is significantly better than other algorithms, and the infection size of the top-10 influential nodes of the CC algorithm is relatively smaller than that of other algorithms.

### Consistency analysis

**Figure 7:**
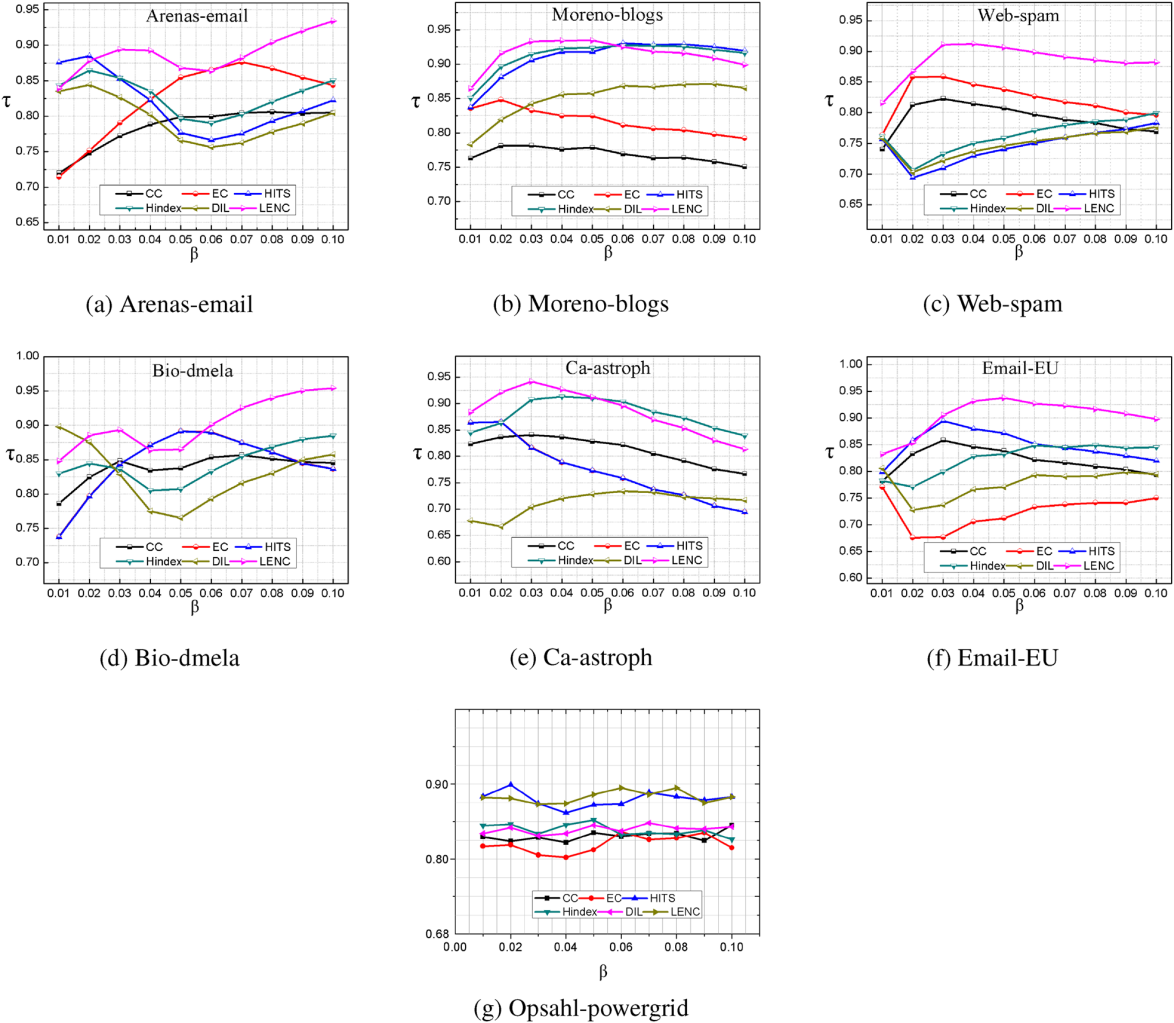
Kendall coefficient comparison under different infection probability conditions.

Kendall coefficient is used to express the similarity and consistency of two sequences^33^. In this experiment, the infection probability of the SIR model is set as [0.01, 0.1]. The infection sequence was obtained through 500 iterations. The higher the Kendall coefficient, the more consistent the algorithm is with the real ranking result, as shown in Fig. 7. In Arena-Email and Bio-dmela networks, the kendall correlation coefficient between the LENC algorithm and SIR model is higher significantly. When the infection probability is greater than 0.06, the effect of the proposed algorithm and SIR model is relatively consistent, which proves that the evaluation results of the LENC algorithm are accurate. In Moreno-blogs and Ca-astroph networks, The Kendall coefficient of the LENC algorithm is the maximum when $\beta \le 0.05$, and then drops to the same value as other algorithms when $\beta >0.05$, but it still has certain advantages, indicating that the algorithm can accurately identify influential nodes in the network. In the Bio-dmela and Email-EU networks, the Kendall coefficient of the LENC algorithm is the largest, indicating that the recognition accuracy of the LENC algorithm is high. In summary, the ranking results of the LENC algorithm are highly consistent with the results of the SIR model, which verifies the accuracy of the algorithm. In the Opsahl-powergrid network, the Kendall coefficient of the LENC and HITS algorithm has the highest value, which is significantly better than other algorithms.

## Discussion

The paper mainly introduces the model construction process of identifying influential nodes based on the entropy of the node based on the weight distribution of the edges connected to it. Introduces the time complexity of the model and the node influence evaluation process, and selects eight real-world networks with different network structure attributes. The experimental verification is carried out in four aspects: case test, correlation analysis, transmission capacity, and consistency analysis. The experiment verifies that the proposed algorithm LENC has obvious advantages. However, when calculating the influence of nodes, to control the time complexity of calculation cost, only the influence of first-order and second-order edges of the nodes are considered, and the accuracy of node influence ranking still has a lot of room for improvement. We will further improve the algorithm in future work.
